# Diagnosis: Melanoderma after Hematopoietic Stem Cell Transplantation

**DOI:** 10.4274/tjh.2014.0475

**Published:** 2015-12-03

**Authors:** Şule Ünal, İlhan Tezcan, Şafak Güçer, Meryem Seda Boyraz, Deniz Çağdaş, Duygu Uçkan Çetinkaya

**Affiliations:** 1 Hacettepe University Faculty of Medicine, Division of Pediatric Hematology, Ankara, Turkey; 2 Hacettepe University Faculty of Medicine, Division of Immunology, Ankara, Turkey; 3 Hacettepe University Faculty of Medicine, Division of Pediatric Pathology, Ankara, Turkey; 4 Hacettepe University Faculty of Medicine, Department of Pediatrics, Ankara, Turkey

**Keywords:** Hematopoietic stem cell transplantation, Melanoderma, Skin findings, SCID

## QUIZ IN HEMATOLOGY

**Figure 1:**
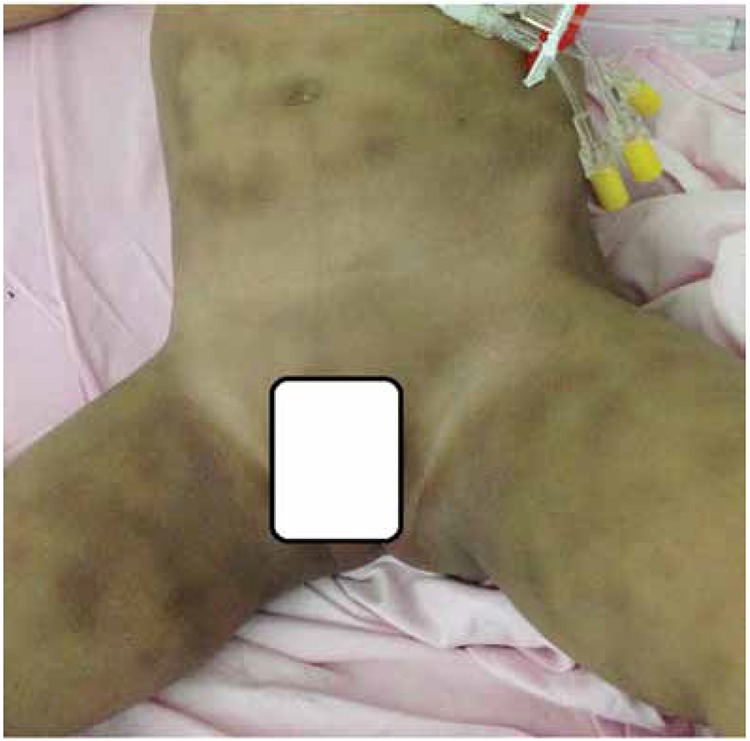
Patchy melanoderma lesions mimicking ecchymoses in the legs, trunk.

**Figure 2:**
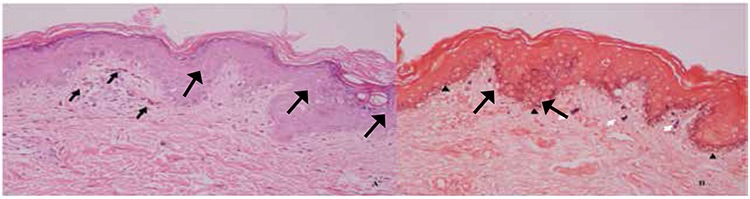
A) Skin. A slight perivascular infiltration of mononuclear inflammatory cells and dermal melanophages (arrows) are seen (HEx200). B) An increment in melanin pigment in basal keratinocytes (arrow heads) and dermal melanophages (white arrows) are highlighted by Fontana-Masson stain (x200).

An 8-month-oldboy diagnosed with T-B+NK- SCID underwent peripheral blood hematopoietic stem cell transplantation (HSCT) from MSD without conditioning. However, he developed pancytopenia and became transfusion dependent by post-transplant 2nd month. Bone marrow aspiration/biopsy revealed an aplastic marrow with 98% donor chimerism. With a diagnosis of T-cell engraftment of the donor but no engraftment of the other lineages, a 2nd HSCT with conditioning (BU/FLU/ATG) was performed at post-transplant +23rd month from the same donor. Due to hyperferritinemia pre-transplant desferoxamine was given. On post-transplant day +2, he developed hyperpigmented patches (Figure 1). Platelet count was 22x109/L and aPTT and PT were normal. Platelet transfusion was given; however the lesions did not subside with the expected color change of ecchymoses. Skin biopsy from medial thigh was obtained (Figure 2).

Generalized hyperpigmentation, after conditioning, is a common finding after HSCT [1]. However, in our patient the lesions were patchy. There are few reports of melanoderma [1,2] after HSCT and in one, melanoderma was reported as a finding of chronic GvHD [2]. Based on the absence of clinical signs of GvHD and lack of typical histological evidence, the melanoderma in our patient was attributed to drugs used in conditioning. The transfusional iron loading may cause a generalized darkening of the skin; however in our patient the lesions were patchy and developed just after completion of the conditioning regimen and subsequent stem cell infusion. The patient did not develop acute or chronic GvHD signs throughout the follow-up. The lesions’ color faded after engraftment gradually, although did not disappear totally. Currently, the patient is alive at post-HSCT 6th month.
